# OseIF3h Regulates Plant Growth and Pollen Development at Translational Level Presumably through Interaction with OsMTA2

**DOI:** 10.3390/plants10061101

**Published:** 2021-05-30

**Authors:** Yuqing Huang, Peng Zheng, Xuejiao Liu, Hao Chen, Jumin Tu

**Affiliations:** Institute of Crop Science, Zhejiang University, Yu-Hang-Tang Road No 866, Hangzhou 310058, China; isco@zju.edu.cn (Y.H.); 11716009@zju.edu.cn (P.Z.); liuxuejiao1229@zju.edu.cn (X.L.); haochen@zju.edu.cn (H.C.)

**Keywords:** OseIF3h, OsMTA2, RNA modification, plant growth, pollen development

## Abstract

The initiation stage of protein biosynthesis is a sophisticated process tightly regulated by numerous initiation factors and their associated components. However, the mechanism underlying translation initiation has not been completely understood in rice. Here, we showed knock-out mutation of the rice eukaryotic translation initiation factor 3 subunit h (OseIF3h) resulted in plant growth retardation and seed-setting rate reduction as compared to the wild type. Further investigation demonstrated an interaction between OseIF3h and OsMTA2 (mRNA adenosine methylase 2), a rice homolog of METTL3 (methyltransferase-like 3) in mammals, which provided new insight into how N6-methyladenosine (m6A) modification of messenger RNA (mRNA) is engaged in the translation initiation process in monocot species. Moreover, the RIP-seq (RNA immunoprecipitation sequencing) data suggested that OseIF3h was involved in multiple biological processes, including photosynthesis, cellular metabolic process, precursor metabolites, and energy generation. Therefore, we infer that OseIF3h interacts with OsMTA2 to target a particular subset of genes at translational level, regulating plant growth and pollen development.

## 1. Introduction

Protein synthesis is predominantly determined by many distinguishing factors at the initiation phase instead of the elongation or termination phase, contributing to instant, flexible and precise regulation over global translation [1]. Among these factors, eIF3 is a multicomponent complex and ubiquitously presents in all eukaryotic organisms. In mammals, eIF3 comprises five constituting subunits eIF3a, -3b, -3c, -3i, and -3g, and seven additional non-core subunits eIF3d, -3e, -3f, -3h, -3k, -3l and -3m [2]. In plants, the eIF3 architecture shares a closely similar composition with mammalian eIF3, in which each subunit has its corresponding mammalian homolog based on protein sequence similarity [3]. In recent years, biochemical analysis, structure explorations and interaction mapping investigations suggested that the eIF3 facilitated the assembly of preinitiation complexes and performed as a docking site for charging of ribosomes, mRNAs and other initiation factors [2,4,5]. The eIF3h subunit presenting on the periphery of the eIF3 complex is an evolutionarily conserved 38-kDa MPN (Mpr1-Pad1-N-terminus) domain protein. Proteins harboring this domain can work as members in constituting the COP9 signalosome, the 26S proteasome lid [6]. In mammals, the increased expression of eIF3h was associated with cancers of breast, liver and prostate via altering the selective translation of mRNA engaged in cell growth control [7,8]. In zebrafish, defects in eIF3h caused aberrant development of the eyes and brain, which could be interpreted by a pool of lens development related mRNAs depletion from the *eif3h* mutant polysomes [9]. In Arabidopsis, unfavorable morphology such as retarded postembryonic growth and reduced pollen viability were observed due to the loss of eIF3h [10]. In the fission yeast *Sz. Pombe*, eIF3h depletion caused a severe defect in meiosis, leading to a high frequency in either no spores or incomplete tetrads [11]. Overall, eIF3h functions as a device for translational governance of a certain subset of cellular mRNAs and exerts a profound impact on normal growth and development.

N6-methyladenosine (m6A) is a prevalent nucleotide modification in eukaryotic mRNA and is required for the regulation of translation [12], subcellular localization [13], splicing [14], and local conformation changes of transcripts [15]. In Arabidopsis, mRNA adenosine methylase (MTA), the ortholog of METTL3 known as “N6-methyladenosine writer”, is an enzyme defined by its methyltransferase activity in mammals catalyzing m6A events [16,17]. *OsMTA2* and *OsFIP* are the orthologs of Arabidopsis *MTA* and *FIP37* (FKBP12 interacting protein 37 kD), respectively [18,19]. They interact with each other and play a vital role in mRNA methylation events. Loss of OsMTA2 leads to embryo-lethal phenotype. Similarly, knockout of MTA results in embryo abortion with failure at the globular development stage in Arabidopsis [16]. OsFIP is necessary for sporogenesis and fertility, whereas the progeny plants of its knock-out mutant can be obtained [19]. A significant reduction in m6A levels due to knockdown of MTA resulted in the reduction of apical dominance, defects in floral organ number and size, and an increased number of trichome branches in Arabidopsis [20]. In mammals, a direct functional and physical interaction between METTL3 and eIF3h supports the mRNA looping mechanism for ribosome recycling and translational control [21], since eIF3 is essential for the attachment of the recycled 40S subunits to mRNA to start a new round [22,23]. Although eIF3h and MTA play crucial roles in the regulation of plant growth and development, the functional significance of the interaction between eIF3h and MTA in plants remains elusive.

Concluded above, eIF3h acts as an upstream regulatory component for some specific transcripts at the translational level and coordinates diverse signaling cascades to maintain optimum translation homeostasis. However, whether eIF3h in monocot species have similar functions as in mammals has not been reported yet. To this end, we generated three independent CRISPR knockout mutants of OseIF3h, whose plant height was decreased to 70%, and the seed-setting rate was decreased to 30% as compared to the wild type, indicating that OseIF3h is essential for rice growth and development. Accordingly, we utilized RIP-seq assay, Co-IP assay, subcellular localization analysis to explicate its biological role in rice growth and development regulation.

## 2. Results

### 2.1. Generation and Characterization of oseif3h Mutants

**Figure 1:**
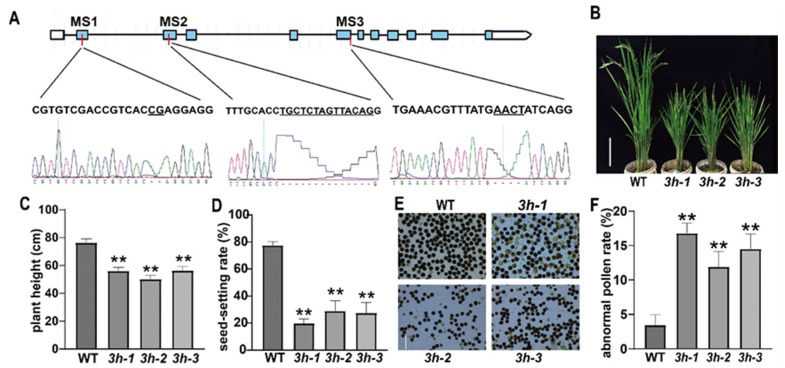
Morphology of *oseif3h* mutants and wide type (WT) rice. (**A**) Schematic diagram of *OseIF3h* gene. Blue boxes denote exons, lines between blue boxes indicate introns. The mutation sites (MS) are marked by the red vertical lines. Sanger sequencing chromatographs of the CRISPR target region showed below. (**B**) Phenotypes of *oseif3h* mutants and WT at heading stage. Bars = 20 cm; (**C**) Plant height of *oseif3h* mutants and WT. *n* = 20. (**D**) Seed-setting rate of *oseif3h* mutants and WT. *n* = 8. (**E**) I2-KI staining of mature pollen grains sampled from oseif3h mutants and WT. Bars = 50 μm. (**F**). Abnormal pollen rate of *oseif3h* mutants and WT. Each microscope field containing more than 50 pollens was calculated. *N* = 5. The significance of difference was evaluated using Student’s t test; ** means *p* < 0.01.

To determine whether the function of OseIF3h bears a close resemblance to that of in Arabidopsis, like regulation role in plant growth and development, we selected three mutation sites (MS) and created three independent mutants, namely 3h-1, 3h-2, and 3h-3 by CRISPR/Cas9 system (Figure 1A). Sequencing analysis showed that these three mutants were caused by the deletion of 2 bp, 14 bp, and 4 bp in exon 1, exon 2, and exon 5 of OseIF3h, respectively (Figure 1A). These non-triple base pair deletions lead to frameshift mutations and premature translation terminations, consequently generating truncated proteins with only 36, 79 and 156 amino acids. Subsequently, expression of OseIF3h was detected at protein level in these mutants using anti-OseIF3h monoclonal antibody. As shown in Figure S1, there was no signal of the full-length protein detected in the three mutants. These results verified that OseIF3h in these three independent mutant lines were indeed knock-out. Further phenotypic analysis indicated that the loss of OseIF3h seriously affected plant growth (Figure 1B). *oseif3h* mutants were about 55 cm in height, which was significantly shorter than that of wild type 77 cm (Figure 1C). Furthermore, severe phenotypic defects were observed on seed-setting rate. Under the same growth conditions, the seed-setting rate of the mutants was merely 20–30%, while that of the wild type was 78.4% (Figure 1D). To figure out whether the reduced seed-setting rate was the consequence of male fertility defect, the pollens were examined by I_2_-KI staining. The results showed that the number of abnormal pollen grains of the mutants was significantly more than that of the wild type (Figure 1E,F). In general, these results indicated that the mutation of *OseIF3h* led to aberrant development in rice, particularly in pollen fertility.

### 2.2. Subcellular Localization of OseIF3h and Its Expression and Polysome Profile Pattern

**Figure 2:**
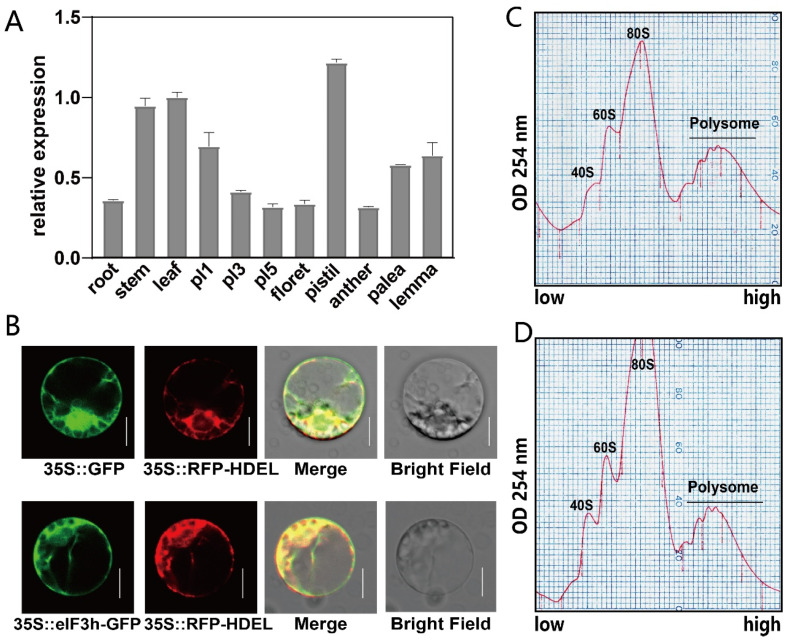
Expression pattern and subcellular localization of OseIF3h and its polysome profile. (**A**) RT-qPCR analysis of *OseIF3h* transcripts in various tissues. Pl1, Pl3, and Pl5 indicate the rice panicle length. Error bars indicate SDs from three biological replicates; (**B**) Subcellular localization of OseIF3h in rice protoplasts (cv Nipponbare background). 35S:OseIF3h-GFP was co-transformed with 35S:RFP-HDEL (an ER marker) into rice shoot protoplasts. Bars = 5 um. Protoplasts transfected with control vector (35S:GFP) have a bright GFP signal distributed in both cytoplasm and nucleus; (**C**,**D**) Representative polysome profiles derived from leaf extracts of WT and *oseif3h-2* mutant, respectively. The position of 40S, 60S, 80S and polysome were marked in the figure, separately. Sedimentation is from low to high.

The expression pattern of *OseIF3h* derived from various tissues revealed that the expression of *OseIF3h* was constitutive, with the highest expression in pistils and leaves (Figure 2A). To detect the subcellular localization of OseIF3h, a GFP (green fluorescent protein) fused protein OseIF3h-GFP driven by the cauliflower mosaic virus 35S promoter was transiently expressed in rice protoplasts. The detected green fluorescence signals of OseIF3h-GFP were largely overlapped with the red fluorescence signals of the endoplasmic reticulum (ER) marker RFP-HDEL (Figure 2B), suggesting an ER-localized feature of OseIF3h.

Previous works have extensively described that eIF3h is one of the subunits of eukaryotic translation initiation factor 3 (eIF3) complex and functions at several steps in the initiation of protein synthesis [24]. To figure out whether the loss of OseIF3h affects translation efficiency, we performed the polysome profiling assay for both mutant and wild type. The results showed that the mutant exhibited a slightly more RNA accumulation in 40S and 60S ribosomal subunit fractions, a significantly higher peak in 80S ribosomal subunit fraction, and a relatively lower peak of polyribosome compared with those in the wild type (Figure 2C,D). This pattern favors the notion that the global translation status was attenuated in the *3h-2* mutant.

### 2.3. OseIF3h Interacts with OsMTA2

**Figure 3:**
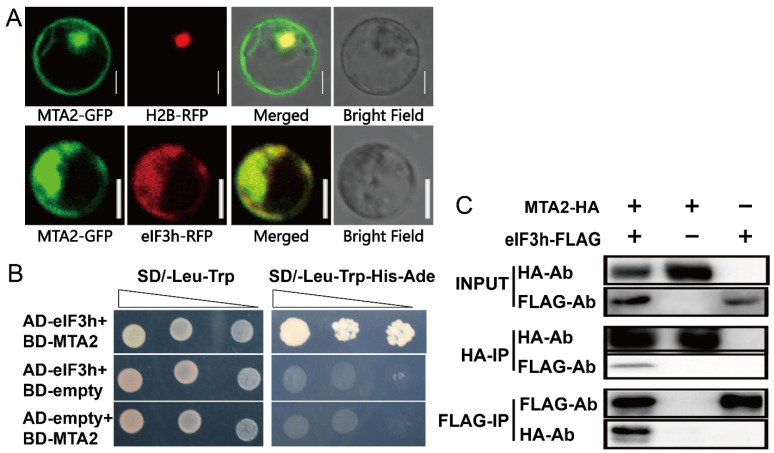
Analysis of interaction between OseIF3h and OsMTA2. (**A**) Subcellular localization of OsMTA2-GFP (upper panel) and co-localization of OseIF3h-RFP with OsMTA2-GFP (lower panel). H2B-RFP is an RFP-labeled nucleus marker. Bar = 5 μm. (**B**) Yeast two-hybrid assay, AD-eIF3h and BD-MTA2 could be grown on SD/-Trp-Leu-His-Ade medium, while the empty vector pGADT7 or pGBKT7 as negative control could not grow. (**C**) Co-IP assay, total protein extracts (input), HA-immunoprecipitation (HA-IP) and FLAG-immunoprecipitation (FLAG-IP) from rice protoplasts transformed with MTA2-HA and eIF3h-FLAG construct or MTA-HA alone construct or eIF3h-FLAG alone construct were analyzed by western blot with anti-HA and anti-FLAG antibodies (HA-Ab and FLAG-Ab).

METTL3 (methyltransferase like 3) is a methyltransferase that catalyzing m6A events and interacts with eIF3h in humans [21,25]. *OsMTA2* is the rice orthologous gene of mammal *METTL3*, and the multiple sequence alignment of *MTA* was shown in Figure S2. To survey whether OsMTA2 interacts with OseIF3h in rice, we firstly examined their co-localization in rice protoplast. The data showed that the signal of GFP-tagged OsMTA2 (OsMTA2-GFP) was diffusely distributed in both nucleus and cytoplasm (Figure 3A) and indeed largely overlapped with eIF3h-RFP signal (Figure 3A). Secondly, we carried out the yeast two-hybrid (Y2H) assay. The DNA segments containing the OsMTA2 and OseIF3h coding sequences were cloned separately into the pGBKT7and pGADT7 vectors to form the prey and bait constructs, respectively. The interaction between OseIF3h and OsMTA2 allows for the yeast proliferating on synthetic defined (SD) medium lacking Ade, His, Trp and Leu. While the empty pGADT7 or pGBKT7 showed no growth (Figure 3B).

Co-immunoprecipitation (Co-IP) assays were performed to substantiate the interaction between OseIF3h and OsMTA2 in rice protoplasts. Three different protein combinations, namely OsMTA2-2×HA and OseIF3h-3×FLAG, OsMTA2-2×HA, or OseIF3h-3×FLAG, were expressed in rice protoplasts through PEG-mediated transformation. Crude protein extracts of cells were pull down by anti-HA antibody or anti-FLAG antibody. By using an anti-FLAG antibody or anti-HA antibody, we detected OsMTA2-2×HA or OseIF3h-3×FLAG in the same immunoprecipitation (Figure 3C). These results confirmed the interaction between OsMTA2 and OseIF3h, suggesting that OseIF3h may be involved in the regulation of protein translation through mRNA methylation modification.

### 2.4. RIP-Seq Data Mapping and Character of OseIF3h Associated Peaks

The knockout of eIF3h in yeast suggested that eIF3h was potentially prone to function as a translational regulator rather than a constitutive component [11]. To identify the substrate candidates of OseIF3h, we performed RNA immunoprecipitation followed by sequencing (RIP-seq) using the floret and leaf mixture as materials. This experiment was conducted with two biological replicates. IP results were validated by western blotting using anti-OseIF3h monoclonal antibody (Figure S3). After removing duplicate reads, approximately 3.6 and 2.5 million reads generated from WT and mutant were uniquely aligned to the rice MUS7 genome (http://rice.plantbiology.msu.edu/, accessed on 28 April 2021). Peak calling was conducted using Piranha software filtering based on a 0.01 FDR (false discovery rate) cutoff. To reduce the effects caused by background noise, we only retained the peaks mutually shared in both replications as high-confidence OseIF3h binding peaks for subsequent analysis.

**Figure 4:**
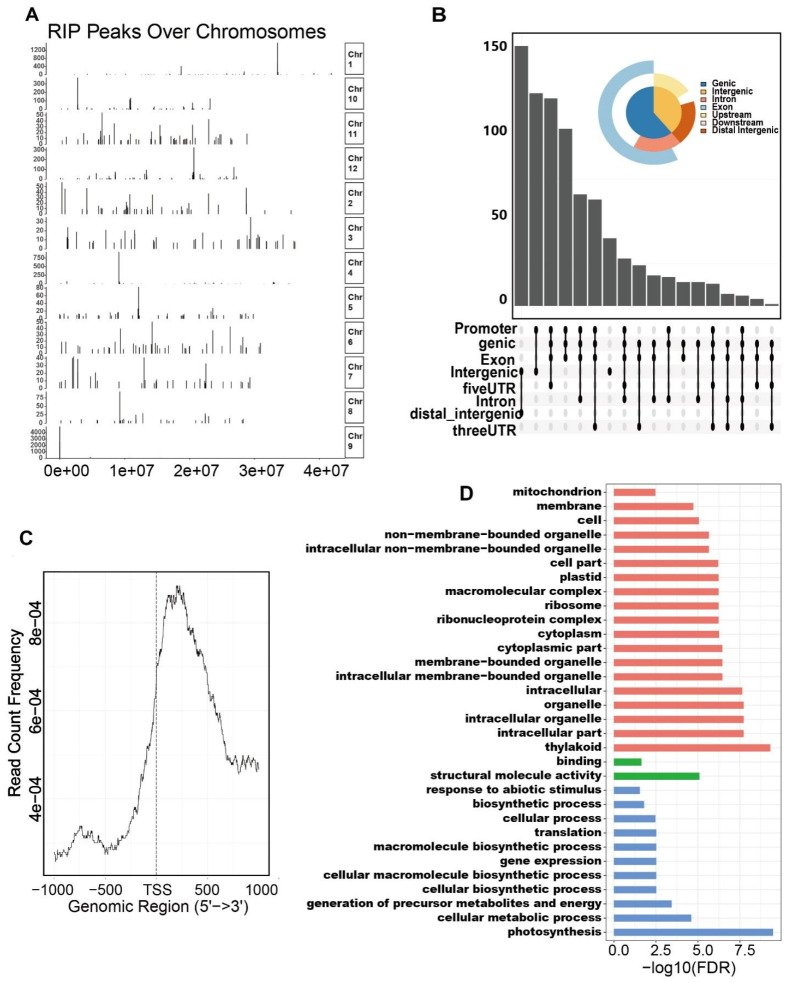
Characterization and distribution of OseIF3h associated peaks over chromosomes. (**A**) Peak location over the whole genome; (**B**) Distribution of peaks in transcript segments divided into 5′UTRs, exons, 3′UTRs, intron and intergenic region; (**C**) Profile of RIP peaks binding to 1kb upstream and 1kb downstream around TSS region; (**D**) Gene ontology (GO) enrichment analysis of gene in the proximity of RIP peaks. Statistical analysis was performed using agriGO online tools.

The coverage plot of putative peaks dispersed in the genomic landscape showed that 823 putative peaks were located to chromosome discriminately and the coverage value differed in a wide range. For example, the peak on chromosome 9 reached a value as high as 4000, which was in sharp contrast to the low value in other chromosomes (Figure 4A, Table S2). The annotation and distribution of peaks were parsed by R script commenced with the bed-format peak file. The position coordinate of each putative peak determined by the peak median was assigned to one of the five non-overlapping transcript sections: 5′-UTRs, exons, 3′-UTRs, intron and intergenic regions. The data revealed that most of the peaks were detected in exons (Figure 4B), indicating that the peak distribution was not random.

To map the precise location for the binding region of OseIF3h, we plotted the count number profile over upstream and downstream of Transcription Starting Site (TSS, −1000 bp to 1000 bp). The accumulation peaks were found to be preferentially enriched in the downstream side of TSS (Figure 4C).To illustrate how OseIF3h exerts its roles at the translational level to regulate rice growth and development, we performed gene ontology enrichment (GO) analysis. The results demonstrated that the candidate genes with false discovery rate (FDR) less than 0.01 were mainly involved in several biological processes, including photosynthesis, cellular metabolic process, generation of precursor metabolites and energy (Figure 4D). Consistent with the biological function, cellular component enrichment analysis revealed strong enrichment in thylakoid (Figure 4D).

## 3. Discussion

### 3.1. OseIF3h Is Required for Plant Growth and Pollen Development in Rice

In the fission yeast *Sz. Pombe*, eIF3h depletion caused a severe defect in meiosis, leading to a very high frequency in either no spores or incomplete tetrads [11]. *ateif3h* insertion mutant has severe phenotype defects in postembryonic growth, female fertility, and pollen vigor that jointly lead to seedlings lethality [10]. However, the roles of eIF3h in monocot plants remain unknown. In the present study, we found that the mutation of *OseIF3h* gene severely affects plant growth at vegetative stage and pollen fertility at reproductive stage and thereby results in a significantly reduced seed-setting rate. These results provide evidence for an indispensable role of OseIF3h in rice.

What confused us is that the high pollen I_2_-KI staining rates of *oseif3h* mutants were disproportional to their considerably low seed setting rates (Figure 1C). However, we could not exclude the possibility of abnormal microgametophyte development merely through I2-KI staining method. It is well known that pollen development comprises two successive stages, namely microsporogenesis and microgametogenesis. Microsporogenesis commences with the formation of pollen mother cells followed by meiosis to produce unicellular microspores. Subsequently, the released microspores undergo two rounds of mitosis during microgametogenesis [26,27]. During mitosis I, the unicellular microspore divides asymmetrically to form bicellular pollen with a small generative cell and a large vegetative cell [26,27]. The generative cell subsequently undergoes the second mitosis to produce two sperm cells wrapped within the vegetative cell, thus leading to the formation of mature tricellular pollen [26,27]. This entire development procedure ensures double fertilization to produce the embryo and endosperm [26,27]. Defects in any of these developmental events can probably lead to pollen abortion. Cytological analysis of another development in rice by Zhang et al. revealed that gametophyte abortion is mainly caused by pollen development defects in tricellular period, which is usually accompanied by starch accumulation [28]. BT-type male sterile line, widely utilized for generating japonica hybrids, is a typical gametophytic male sterile line characterizing with stained abortion pollens [29]. On the basis of the above results, we speculate that the pollen of *oseif3h* mutants may have defects in microgametogenesis stage and thus lead to the significant decrease in seed setting rate.

### 3.2. OseIF3h Involves in the Regulation of Protein Translation Presumably through mRNA Methylation

In the present study, the polysome profiling assay and the ER subcellular localization of OseIF3h confirmed the roles of OseIF3h in protein synthesis and regulation. Considering that protein synthesis is a major regulatory step of gene expression in different physiological processes including development [30], it is reasonable for us to believe that OseIF3h, a subunit of rice translation initiation factor 3, plays a role in the whole developmental processes of rice. In fact, the constitutive expression pattern of *OseIF3h* also proved this point (Figure 2A).

In mammals, METTL3 enhanced the translation of oncogene transcripts modified by m6A near the stop codon by associating with eIF3h to form a loop and accelerate ribosome recycling [21,31]. METTL3 depletion caused an accumulation in the 80S ribosome peak in polysome profiling assay and had a trivial effect on steady-state mRNA loading [21]. Corresponding to this, our results demonstrated that OsMTA2, the ortholog of METTL3 in rice, interacts with OseIF3h confirmed by yeast two-hybrid assay and Co-IP assay, raising the possibility that there is a similar modulating mechanism in rice. As shown in Figure 2D,Figure 2E, the knockout mutant *3h-2* displayed a similar polysome profiling pattern as in mammalian METTL3 depletion, suggesting tight cooperation between OseIF3h and OsMTA2 in translational regulation.

### 3.3. OseIF3h Is Required for Photosynthetic Performance

RIP-seq is reported to be a robust technology in identifying RNA candidates of ribonucleoproteins that are not physically direct bound [32]. This approach is amenable for the study of multisubunit ribonucleoprotein like OseIF3. RIP-seq encompasses whole transcript-scale binding information rather than the site-degree resolution of CLIP-seq methods. Here, the high background noise, presumably as the consequence of the FA crosslinking, hampers the peak calling accuracy [33]. Therefore, only peaks overlap in two biological replicates are regarded as true peaks to conduct GO analysis.

GO enrichment analysis presented that the primary target genes of OseIF3h are relative to the photosynthesis process. Consistent with this result, the expression pattern of OseIF3h showed high abundance in green tissues, including leaf, stem, lemma and palea. Photosynthesis occurs in chloroplasts, which assist in storing and harvesting required substances for energy production to support plant growth and development [34]. Likewise, cellular component enrichment analysis also reveals a strong enrichment in thylakoid. These evidences facilitate interpreting the *oseif3h* mutant phenotypes observed in this study.

OseIF3h was reported to interact with AET1, a tRNA^His^ guanylyltransferase that catalyzes the modification reaction of pre-tRNA^His^ and facilitates the normal growth under high-temperature conditions through translation regulation in rice [35]. Notably, our GO enrichment analysis indicated that OseIF3h respond to abiotic stimulus, such as drought, heat, and salt stresses. Deep research in this field will provide new insights into how plants can cope with abiotic stress from translational level.

Taking together, this study reveals the regulation role of OseIF3h in protein translation initiation, which is presumably realized through mRNA methylation. In addition, RIP-Seq analysis performed in this study also showed that OseIF3h was involved in the regulation of photosynthesis, which may explain the reason why OseIF3h knockout mutants exhibit short plant height and decreased seed-setting rate.

## 4. Materials and Methods

### 4.1. Genetic Material

Rice cultivar *Oryza sativa* L. ssp. *Japonica* ‘Nipponbare’ was used as a recipient for all permanent and transient transformations conducted in this study.

### 4.2. Generation of oseif3h Mutants

To create *oseif3h* mutant, we designed sgRNA using the online tool (http://crispr.hzau.edu.cn/CRISPR/, accessed on 28 April 2021). The sgRNA selection criterion is in close proximity to the transcription start site with high evaluation score. To generate CRISPR/Cas9 binary constructs, we inserted the sgRNA driven by OsU6a pol III promoters into the pCAMBIA1300-based Cas9 vector via a Golden Gate ligation method. The sequencing confirmed plasmids were then transformed into Nipponbare callus to create a stable transgenic line by *Agrobacterium*-mediated transformation as previously described [36]. Subsequently, the transgenic lines were screened based on the hygromycin resistance and direct sanger-sequencing of the target-containing amplicons. Primers used in this research are listed in Table S1.

### 4.3. Yeast Two-Hybrid Assays

Yeast two-hybrid assays were performed using yeast AH109 nutrition deficient strain. The OsMTA2 and OseIF3h coding sequences were amplified, respectively. PCR products were then cloned into pGBKT7 and pGADT7 (Clontech) vectors to form bait and prey constructs using the ClonExpress Ultra One Step Cloning Kit (Vazyme, Nanjing, China). All constructs were confirmed by sequencing. Empty vectors were used as negative controls. All bait and prey construct pairs were transformed into the yeast AH109 strain via PEG/LiCl mediated transformation methods. The yeast cells were cultured at 28 °C on SD/-Trp-Leu for successful transformation testing before they are dotted onto SD/Trp-Leu-His-Ade medium plates for interaction evaluation.

### 4.4. Transient Expression in Protoplast and Co-IP Assay

Rice protoplasts were prepared and transfected as previously described [37]. The protoplasts co-transformed with 20 μg plasmid (10 μg for each construct) were harvested by centrifugation at 5000 rpm for 30 s after incubation for 12 h. Approximate 2 × 10^6^ protoplasts were homogenized in IP buffer [25 mM Tris-HCl, pH 7.5, 150 mM NaCl, 1% Triton X-100, 1 mM EDTA, and Protease Inhibitor Cocktail (Roche, Palo Alto, NJ, USA)] and incubated for 30 min on ice with an intermittent vortex to ensure complete lysis. The mixture was centrifuged at 20,000× *g* for 10 min at 4 °C to discard debris. Thirty microliters of Protein A/G Agarose Beads (Abmart, Shanghai, China) were added to get the pre-clean extract by incubation for 3 h at 4 °C with gentle rotation. One microgram of antibodies were added to the pre-clean extract followed by incubation at 4 °C overnight. Thirty microliters of pre-equilibrated Protein A/G Agarose Beads were added and incubated for a further 3 h at 4 °C. The beads were collected by centrifugation at 100× *g* for 30 s at 4 °C and then washed five times with pre-chilled IP buffer. The proteins were eluted by boiling in SDS-PAGE loading buffer for 5 min before detection by western blot. The rat monoclonal anti-HA antibody was bought from Roche (11867423001) and the mouse monoclonal anti-FLAG antibody were from MBL International (M185-3L).

### 4.5. Polysome Profiling Assays

Polysome profiling assays were implemented as previously described with some modifications [38]. Ribosome was extracted from the leaves by grounding in liquid nitrogen. The pulverized tissue were homogenized in pre-chilled ribosome extraction buffer (0.2 M Tris-HCl pH 9.0, 0.2 M KCl, 0.035 M MgCl_2_, 0.025M EGTA, 50 μg/mL cycloheximide, 50 μg/mL chloramphenicol, 1% Triton X-100, 5 mM DTT, and Protease Inhibitor Cocktail) for 30 min on ice with gentle mixing to ensure thoroughly lysis. The mixture subsequently filtered through two layers of sterile microcloth into an RNA-free tube. The extracts were loaded gently and slowly on top of the sucrose cushion (0.4 M Tris pH 9.0, 0.2 M KCl, 0.005 M EGTA, 0.035 M MgCl_2_, 1.75 M Sucrose) to avoid mixing the sample with sucrose cushion and centrifuged at 4 °C, 170,000× *g* (Beckman Ti70 rotor) for 3 h to get the ribosome pellets. The resulting pellet were resuspended in 700 μL of resuspension buffer (0.2 M Tris–HCl pH 9.0, 0.2 mM KCl, 35 mM MgCl_2_, 25 mM EGTA, 100 μg/mL chloramphenicol, and 50 μg/mL cycloheximide) and incubated on ice for 30 min. The ribosome-containing solutions were loaded onto an 11-mL 20–60% continuous sucrose density gradient and centrifuged at 237,000× *g* for 4 h at 4 °C (Beckman SW 40 Ti rotor). After ultracentrifugation, the absorbance of 254 nm was continuously monitored to detect the position of the different compositions using a Teledyne Isco Density Gradient Fractionation System (Teledyne Isco, Lincoln, NE, USA) with spectrophotometric detection device.

### 4.6. RIP-Seq and Data Analysis

RIP assays were implemented as previously described with some modifications [33]. Briefly, RNP complex in mature florets and leaves captured by formaldehyde crosslinking were ground in liquid nitrogen and the supernatant was incubated overnight at 4 °C with 10 μg eIF3h-antibody and further incubated with protein A/G Dynabeads for 2 h at 4 °C. The beads were washed with lysis buffer, high-salt buffer every two times after removing the supernatants. Suspend the beads in fresh elution buffer (50 mM Tris 8.0, 10 mM EDTA and 1% SDS). The RNA was purified with Trizol reagent (Life Technologies, Carlsbad, CA, USA). The cDNA libraries were prepared by the Illumina ScriptSeq™ v2 RNA-Seq Library Preparation Kit (Epicentre, Charlotte, NC, USA), according to the manufacturer’s instructions. Finally, the libraries were applied to the Illumina HiSeq X Ten system for 150 nt paired-end sequencing. The seq data have been deposited into the NCBI Short Read Archive (SRA) under accession number PRJNA725322.

The data were analyzed in the pipeline with default settings except for customer parameters. Briefly, the Bowtie2 [39] was run to align the WT and mutant samples’ clean reads to the MUS7 rice reference file [40]. Piranha, a peak-calling tool based on the zero-truncated negative binomial regression model, was used for peak calling with default settings [41]. The parameter of bin size was set to 300 considering the computer configuration. Peaks were considered if FDR < 0.01 and stored in a bed-format file for subsequent annotation. Peaks annotation and plots were utilized in the R/Bioconductor ChIPSeeker package [42]. Gene ontology (GO) enrichment analysis of genes bearing RIP peaks was performed using agriGO online tools [43].

### 4.7. I_2_-KI Staining for Pollen Fertility

Three spikelets from each plant (three plants for each material) were collected for pollen fertility experiments. Pollen grains at the mature stage were stained with 1% iodine-potassium iodide solution (I_2_-KI) solution and photographed using a Nikon DS-Ri2 microscope. Five fields of each sample with at least 50 pollen grains per visual field were counted. Abnormally sized and unstained pollens were counted as “abnormal”. The pollen number were counted using ImageJ software.

## Data Availability

Data is contained within the article or supplementary material.

## Supplementary Materials

The following are available online at https://www.mdpi.com/article/10.3390/plants10061101/s1, Figure S1. Western blot analysis with eIF3h antibody probe against total proteins extracts prepared from the *3h-1, 3h-2, 3h-3* mutants and WT. Figure S2 The RIP sample probed against anti-eIF3h antibody. Figure S3 Sequence alignments of MTA2 protein in *Arabidopsis, Oryza sativa, Homo sapiens*. Table S1. Primers used in this study. Table S2 Annotation of the OseIF3h binding peaks.
