# Supplementary material for: OseIF3h Regulates Plant Growth and Pollen Development at Translational Level Presumably through Interaction with OsMTA2
Source: Plants (Basel). 2021 May 30;10(6):1101. doi: 10.3390/plants10061101 (PMC8228589; doi:10.3390/plants10061101)
Supplement: Supplementary file 1 [file plants-10-01101-s001.zip › plants-1220055-supplementary/SM/Supplementary materials.pdf]

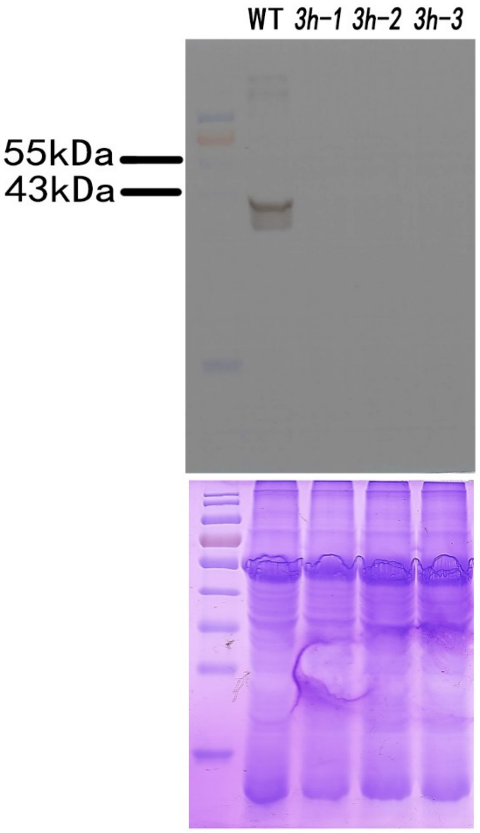

Figure S1 Detection of OseIF3h in WT and mutant lines. Western blot analysis with anti-eIF3h antibody probed against total proteins extracts from the *3h-1,3h-2,3h-3* mutants and WT seedlings (top panel), and the loading control of coomassie Blue staining (bottom panel).

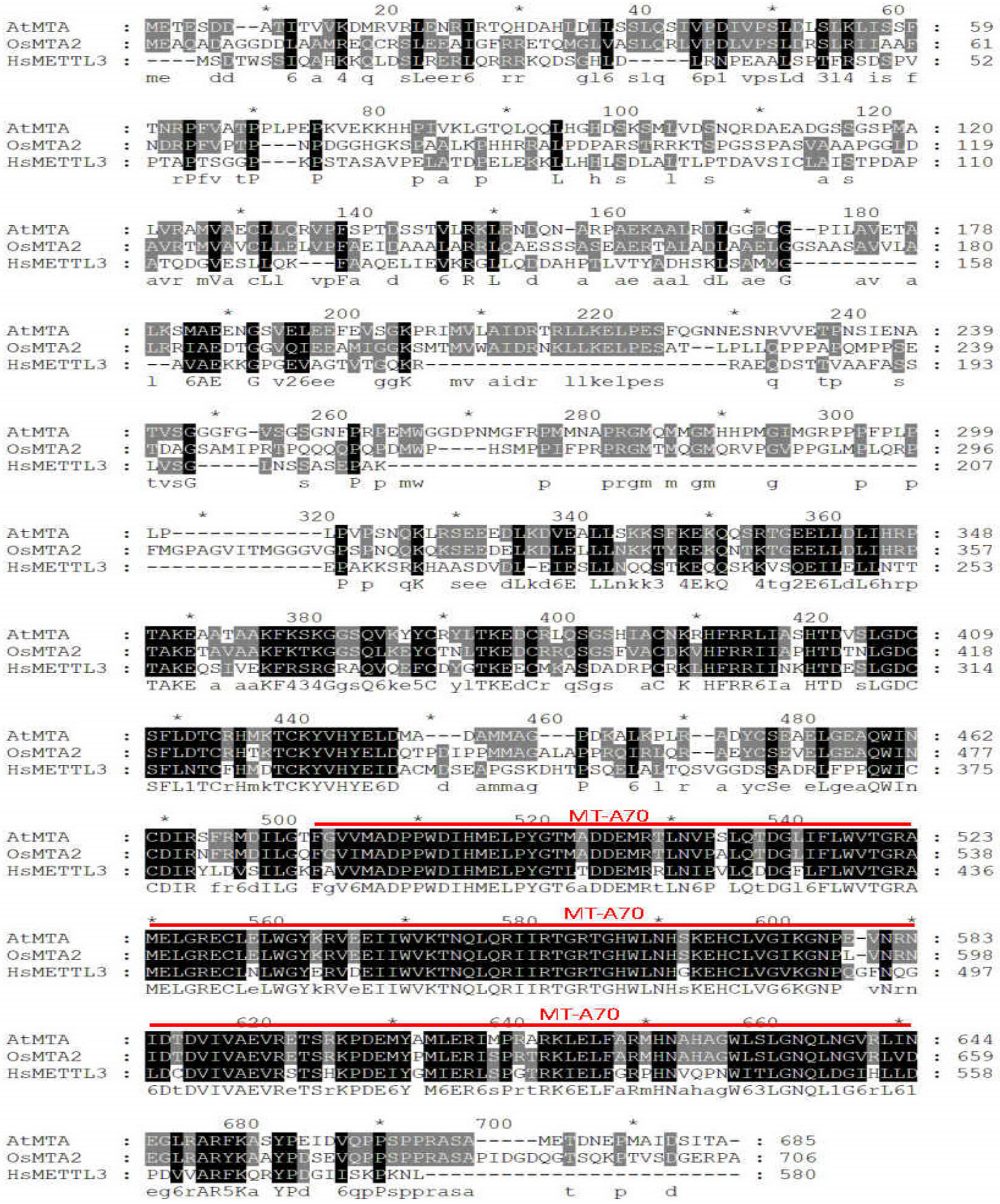

Figure S2. Sequence alignments of MTA2 protein in *Arabidopsis, Oryza sativa, Homo sapiens*.The alignment was obtained by submitting the sequences to ClustalX. Black shading is used to mark identical amino acids. Gray shading is used to mark similar amino acids found among the majority of sequences. The red line indicates the MT-A70 catalyzing domain.

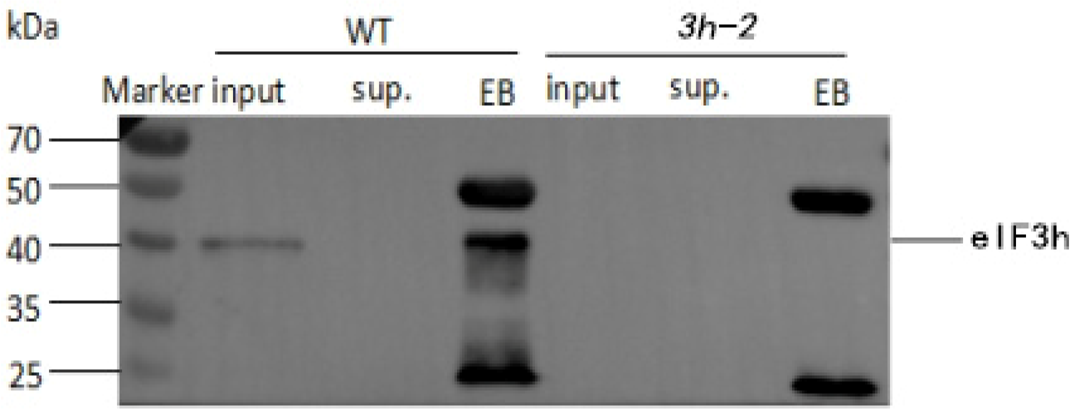

Figure S3. The RIP sample probed against with anti-eIF3h antibody. Immunodetection of the immunoprecipitated protein. Input indicates total protein; sup indicates supernatant; EB indicates Elution buffer.

Table S1. The primers used in this study

Prime name	PRIMER SEQUENCE (5′→3′)	Use
GP1098-eif3h1-F	ACTTCATCCCAAGCGCAAGT	mutants screen
GP1098-eif3h1-R	CACACGAGCGAATCTGACCT	mutants screen
GP1099-eif3h2-F	CCTCCCTCAAGCACCTCATC	mutants screen
GP1099-eif3h2-R	GGCCAGCCGCATTTTTCTTT	mutants screen
GP1100-eif3h3-F	TGTTCTGCATGCAACATCGC	mutants screen
GP1100-eif3h3-R	AGCTGTAGCCTGTTCTGCTT	mutants screen
3hygdl2750F	ATCAGAGCAGAACAAGTTCCAAT	eIF3h qRT-PCR
3hygdl2862F	CAGCAGCTTTTCTTGCCATA	eIF3h qRT-PCR
3hygdl1790F	TCAAGACAGCAGTCACAGCA	eIF3h qRT-PCR
3hygdl1862F	CAGCAGCTTTTCTTGCCATA	eIF3h qRT-PCR
qpcr-actinF	TGAAGATCAAGGTGGTCGCC	actin qRT-PCR
qpcr-actinR	CCTTGGCAATCCACATCTGC	actin qRT-PCR
qpcr-ubqF	ACCACTTCGACCGCCACTACT	Ubiquitin qRT-PCR
qpcr-ubqR	ACGCCTAAGCCTGCTGGTT	Ubiquitin qRT-PCR
xbateeif3hF	GGGGTCTAGAATGGCGAATCCGGCAGCAGCA	pm999 eIF3-GFP construct
xbaI3hR	GGGGTCTAGAGTCCTCCTGCAAGGCCTTCA	pm999 eIF3-GFP construct
3hgfpinfusionF	AACACGGGGGACTCTAGATGGCGAATCCGGCAGCA	1300 eIF3-GFP construct
3hgfpinfusionF	TGTTGGATCCTCTAGGTCCTCCTGCAAGGCCTT	1300 eIF3-GFP construct
mta2HAF	CCCTTGCTCCGTGGATCCATGGAGGCGCAGGCGGAC	HBT MTA2-HA construct
mta2HAR	GTCGTATGGGTAAGGCCTGGCAGGCCTCTCGCCGTC	HBT MTA2-HA construct
OSMTA2BKECORIF	GCCATGGAGGCCGAATTCGCAGCCGCAACAGCAATGGA	pGBKT7 MTA2 construct
OSMTA2BKECORIR	ACGGATCCCCGGGAATTCGGTGCCGACTGCTTCACATC	pGBKT7 MTA2 construct
